# The performance of patients with cerebral microbleeds in different cognitive tests: A cross-sectional study

**DOI:** 10.3389/fnagi.2023.1114426

**Published:** 2023-04-11

**Authors:** Xuanting Li, Shuna Yang, Yue Li, Wei Qin, Lei Yang, Junliang Yuan, Wenli Hu

**Affiliations:** ^1^Department of Neurology, Beijing Chaoyang Hospital, Capital Medical University, Beijing, China; ^2^Department of Neurology, NHC Key Laboratory of Mental Health (Peking University), National Clinical Research Center for Mental Disorders, Peking University Sixth Hospital, Peking University Institute of Mental Health, Beijing, China

**Keywords:** cerebral microbleed, cerebral small vessel disease, cognitive impairment, cognitive test, neuroimaging

## Abstract

**Background:**

The clinical features and pathological process of cerebral microbleed (CMB)-related cognitive impairment are hot topics of cerebral small vessel disease (CSVD). However, how to choose a more suitable cognitive assessment battery for CMB patients is still an urgent issue to be solved. This study aimed to analyze the performance of CMB patients on different cognitive tests.

**Methods:**

This study was designed as a cross-sectional study. The five main markers of CSVD (including the CMB, white matter hyperintensities, perivascular spaces, lacunes and brain atrophy) were assessed according to magnetic resonance imaging. The burden of CMB was categorized into four grades based on the total number of lesions. Cognitive function was assessed by Mini-Mental State Examination (MMSE), Trail-Making Test (TMT, Part A and Part B), Stroop color-word test (Stroop test, Part A, B and C), Verbal Fluency Test (VF, animal), Digit-Symbol Substitution Test (DSST), Digit Cancellation Test (DCT) and Maze. Multiple linear regression analysis was conducted to analyze the association between CMB and cognitive findings.

**Results:**

A total of 563 participants (median age of 69 years) were enrolled in this study, including 218 (38.7%) CMB patients. CMB patients showed worse performance than non-CMB subjects in each cognitive test. Correlation analysis indicated the total number of CMB lesions had positive correlations with the time of TMT, Maze and Stroop test, and negative correlations with the performance of MMSE, VF, DSST, and DCT. After the adjustment for all the potential confounders by linear regression, the CMB burden grade was correlated with the performance of VF, Stroop test C, Maze and DCT.

**Conclusion:**

The presence of CMB lesions was associated with much worse cognitive performances. In VF, Stroop test C, Maze and DCT, the correlations between CMB severity and assessment results were more significant. Our study further confirmed that the attention/executive function domain was the most commonly evaluated in CMB, which provided a picture of the most utilized tools to analyze the prognostic and diagnostic value in CMB.

## Introduction

Increased life expectancy poses a huge public health challenge to society, and advanced age is also associated with an increased risk of dementia. The cerebral microbleed (CMB) is one of the important neuroimaging markers for cerebral small vessel disease (CSVD), and its’ prevalence increases with age (Poels et al., 2011). Besides, more and more studies have found that CMB can lead to cognitive impairment and is an independent risk factor for dementia (Poels et al., 2012; Akoudad et al., 2016; Ding et al., 2017).

Studies have found that CMB lesions can coexist with some neurodegenerative diseases such as Parkinson’s disease and Alzheimer’s disease (AD), and may be related to cognitive and motor dysfunction (Sparacia et al., 2017; Chiu et al., 2018; Tsai et al., 2021). CMB lesions have similar pathological manifestations (β-amyloid accumulation) as AD, while the feature of the cognitive impairment may be different. CMB may be useful to expound the overlap between cerebrovascular and neurodegenerative mechanisms underlying cognitive decline and dementia (Akoudad et al., 2016).

To date, it is still a hot issue now for the specific characteristic of the damage to different cognitive domains about CMB numbers and location. Previous studies have found CMB patients have more serious impairments in special cognitive domains such as attention, executive function, processing speed and memory (van Norden et al., 2011; Poels et al., 2012; Meier et al., 2014; Qian et al., 2021; Nannoni et al., 2022). However, the performance of CMB patients on different cognitive domain tests has been poorly studied.

The purpose of this study was to describe the performance of CMB patients in different cognitive domains and find out which tests are closely related to the CMB burden independent of other CSVD markers. Our study may be helpful to design a battery of neuropsychology assessments, which is more suitable for the early identification of CMB-related cognitive impairment and the observation of CMB burden in clinical practice.

## Materials and methods

### Study population

This was a cross-sectional study. Subjects for the physical examination were recruited from the department of Neurology at Beijing Chaoyang Hospital, Capital Medical University from January 2018 to December 2021. This study was reviewed and approved by the Ethics Committee of Beijing Chaoyang Hospital, Capital Medical University. Written informed consent was obtained from each eligible subject.

Participants aged 40 years or older with available clinical data and brain magnetic resonance imaging (MRI) were included in the study. The exclusion criteria included: (1) acute cerebrovascular diseases; (2) history of the massive relevant cerebral infarction or cerebral hemorrhage with obvious neurological sequelae; (3) brain damage caused by neurodegeneration, infection, inflammation, trauma, tumor, poisoning and metabolic disease; (4) severe psychiatric disorders; (5) severe organ insufficiency; (6) severe visual or hearing impairment; (7) taking cognitive-affecting drugs within 24 h, such as sedative-hypnotic drugs, acetylcholine-esterase inhibitors and so on (van Norden et al., 2011; Wang et al., 2017); and (8) incomplete data or brain MRI with poor quality.

### Assessment of neuroimaging

Magnetic resonance imaging was performed on the 3.0-T MRI scanner (Prisma; Siemens AG, Erlangen, Germany) in the department of Radiology at Beijing Chaoyang Hospital. The standardized sequences included T1-weighted imaging (repetition time = 2,000.0 ms, echo time = 9.2 ms, slice thickness = 5.0 mm, and field of view = 220 × 220 mm^2^), T2-weighted imaging (repetition time = 4,500.0 ms, echo time = 84.0 ms, slice thickness = 5.0 mm, and field of view = 220 × 220 mm^2^), fluid-attenuated inversion recovery (repetition time = 8,000.0 ms, echo time = 86.0 ms, slice thickness = 5.0 mm, and field of view = 199 × 220 mm^2^), diffusion weighted imaging (repetition time = 3,300.0 ms, echo time = 91.0 ms, slice thickness = 5.0 mm, field of view = 230 × 230 mm^2^, and *b* = 0 and 1,000 s/mm^2^), and susceptibility weighted imaging (repetition time = 27.0 ms, echo time = 20.0 ms, slice thickness = 3.2 mm, and field of view = 172 × 230 mm^2^).

Imaging markers of CSVD were defined according to STandards for ReportIng Vascular changes on nEuroimaging criteria described previously (Wardlaw et al., 2013). CMB was evaluated according to Microbleed Anatomical Rating Scale (Gregoire et al., 2009). The total number of CMB lesions was recorded. And, the burden of CMB was categorized into four grades: non-CMB, 1 CMB, 2–4 CMBs, 5–9 CMBs, and ≥10 CMBs. The white matter hyperintensity (WMH) in periventricular and deep areas was graded using the Fazekas scale (range 0 to 3, respectively) (Fazekas et al., 1987). The severity of perivascular space (PVS) in basal ganglia (BG) and centrum semiovale was divided into five grades (range 0 to 4, respectively) (Maclullich et al., 2004). Brain atrophy was evaluated using the visual rating scale for posterior atrophy ranging from 0 to 3 (Koedam et al., 2011). The number of subjects with lacune was recorded.

### Assessment of cognitive function

All participants underwent the face-to-face assessment of cognitive function. The selection strategy of cognitive scales was based on the characteristics of vascular cognitive impairment and prior large clinical studies on CSVD-related cognitive dysfunction (Benisty et al., 2009; Akoudad et al., 2016). The seven tests in this study included Mini-Mental State Examination (MMSE), Trail-Making Test (TMT, including Part A and Part B), Stroop Color-Word Test (Stroop test, including Part A, B and C), Verbal Fluency Test (VF, animal), Digit-Symbol Substitution Test (DSST), Digit Cancellation Test (DCT) and Maze. We recorded the time taken to complete the TMT, Stroop test and Maze, as well as the number of correct responses in the VF, DSST, and DCT within the specified time, respectively. The major cognitive domains assessed by these tests include global cognitive function, executive function, processing speed, attention, language, and visuospatial function.

### Assessment of covariates

Basic clinical information of each participant was collected according to medical records and questionnaires, including age, gender, years of education, body mass index, smoking and drinking status, medication use (hypotensive drugs, statin, antiplatelet drugs/anticoagulants, hypoglycemic drugs), serological test (total cholesterol, high-density lipoprotein cholesterol, low-density lipoprotein cholesterol, triglyceride, glycosylated hemoglobin, homocysteine), and medical history (hypertension, diabetes, hyperlipidemia, stroke, transient ischemic attack, and cardiovascular diseases).

### Statistical analysis

Mann–Whitney U test and chi-square test were used to compare the differences between CMB patients and non-CMB subjects. The Kruskal–Wallis H test was used to compare the differences in cognition among subjects with different CMB burden grades. Spearman’s correlation analysis was used for the association between the CMB number (abnormal distribution) and the results of cognitive tests. Multiple linear regression analysis (stepwise method) was used for the relationship between CMB burden grades/CMB lesion number and cognitive function after the confounder adjustment. We performed tests for linear trends based on the variable containing a median value of each CMB burden grade. Statistical analysis was performed using Statistical Product and Service Solutions 26.0. A *p*-value of less than 0.05 was considered significant.

## Result

A total of 563 subjects were included in this study with a median age of 69 years, including 324 (57.5%) males and 218 (38.7%) CMB patients. Compared with non-CMB subjects, the CMB group had more male patients (*p* < 0.001), fewer years of education (*p* = 0.018), higher smoking rate (*p* = 0.001), more people with hypertension (*p* = 0.003) and stroke/TIA (*p* < 0.001), higher rates of taking antihypertensive drugs (*p* = 0.020) and antithrombotic drugs (*p* < 0.001), and higher serum levels of triglyceride (*p* = 0.008) and homocysteine (*p* = 0.001). As for other imaging markers of CSVD, patients with CMB had a higher prevalence of the lacune (*p* < 0.001), more severe WMH (*p* < 0.001) and BG-PVS (*p* < 0.001) than non-CMB subjects. Table 1 shows the clinical characteristics of all enrolled participants.

**TABLE 1:** Demographic data and clinical characteristics.

	Total (*n* = 563)	Non-CMB (*n* = 345)	CMB (*n* = 218)	*p*
Age^a^ [year]	69 (63, 75)	69 (64, 75)	69 (63, 74)	0.685
Men [*n* (%)]	324 (57.5%)	177 (51.3%)	147 (67.4%)	<0.001*
Years of education^a^ [year]	9.0 (9.0, 12.0)	9.0 (9.0, 12.0)	9.0 (8.8, 12.0)	0.018*
**Cardiovascular risk factors/diseases**				
BMI^a^ [kg/m^2^]	25.39 (22.89, 27.64)	25.39 (23.17, 27.55)	25.66 (22.73, 27.68)	0.648
Smoking [*n* (%)]	196 (34.8%)	101 (29.3%)	95 (43.6%)	0.001*
Drinking [*n* (%)]	122 (21.7%)	66 (19.1%)	56 (25.7%)	0.066
Hypertension [*n* (%)]	380 (67.5%)	217 (62.9%)	163 (74.8%)	0.003*
Diabetes mellitus [*n* (%)]	160 (28.4%)	93 (27.0%)	67 (30.7%)	0.333
Cardiovascular diseases [*n* (%)]	112 (19.9%)	75 (21.7%)	37 (17.0%)	0.168
Stroke/TIA [*n* (%)]	124 (22.0%)	48 (13.9%)	76 (34.9%)	<0.001*
Hyperlipidemia [*n* (%)]	173 (30.7%)	101 (29.3%)	72 (33.0%)	0.347
**Medication use [*n* (%)]**				
Hypotensive drugs [*n* (%)]	338 (60.0%)	194 (56.2%)	144 (66.1%)	0.020*
Statin [*n* (%)]	152 (27.0%)	90 (26.1%)	62 (28.4%)	0.540
Antiplatelet drugs/anticoagulants [*n* (%)]	154 (27.4%)	73 (21.2%)	81 (37.2%)	<0.001*
Hypoglycemic drugs [*n* (%)]	154 (27.4%)	90 (26.1%)	64 (29.4%)	0.396
**Serological test**				
CHOL^a^ [mmol/L]	4.42 (3.72, 5.04)	4.41 (3.65, 5.04)	4.42 (3.88, 5.04)	0.347
HDL-C^a^ [mmol/L]	1.10 (0.90, 1.30)	1.14 (0.94, 1.30)	1.08 (0.90, 1.30)	0.093
LDL-C^a^ [mmol/L]	2.70 (2.07, 3.30)	2.70 (1.98, 3.30)	2.70 (2.20, 3.26)	0.397
Triglyceride^a^ [mmol/L]	1.40 (1.03, 1.83)	1.33 (0.96, 1.82)	1.51 (1.13, 1.99)	0.008*
HbA1C^a^ [%]	6.1 (5.7, 6.6)	6.1 (5.7, 6.5)	6.2 (5.6, 6.7)	0.560
Homocysteine^a^ [μmol/L]	14 (11, 16)	13 (11, 16)	15 (11, 18)	<0.001*
**Brain MRI markers**				
Lacune [*n* (%)]	240 (42.6%)	94 (27.2%)	146 (67.0%)	<0.001*
Posterior atrophy [*n* (%)]	301 (53.5%)	177 (51.3%)	124 (56.9%)	0.196
**Fazekas scale^a^**				
Periventricular WMH	1 (1, 2)	1 (1, 2)	2 (1, 2)	<0.001*
Deep WMH	1 (1, 2)	1 (1, 2)	2 (1, 2)	<0.001*
**PVS^a^**				
BG-PVS	1 (1, 2)	1 (1, 2)	2 (1, 3)	<0.001*
CSO-PVS	2 (1, 3)	2 (1, 3)	2 (1, 3)	0.954

BG, basal ganglia; BMI, body mass index; CHOL, total cholesterol; CMB, cerebral microbleed; CSO, centrum semiovale; WMH, white matter hyperintensity; HbA1C, glycosylated hemoglobin; HDL-C, high-density lipoprotein cholesterol; LDL-L, low-density lipoprotein cholesterol; MRI, magnetic resonance imaging; PVS, perivascular space; TIA, transient ischemic attack.

^a^Median (quartiles).

*P < 0.05.

Comparing the results of cognitive tests between the two groups, CMB patients showed significantly worse global cognitive function (MMSE) than the non-CMB group (*p* < 0.001). CMB patients took more time in Maze (*p* = 0.001), TMT (Part A and B, *p* < 0.001) and Stroop test (Part A, B and C; *p* < 0.001), and had fewer correct responses in VF (*p* < 0.001), DSST (*p* < 0.001) and DCT (*p* < 0.001). These results are shown in Table 2.

**TABLE 2:** Comparison of cognitive function between CMB and non-CMB subjects.

	Total (*n* = 563)	Non-CMB (*n* = 345)	CMB (*n* = 218)	*p*
MMSE^a^	28 (26, 29)	28 (27, 29)	27 (24, 29)	<0.001*
VF^a^	17 (14, 21)	18 (15, 21)	16 (12, 19)	<0.001*
TMT A^a^ (s)	64.91 (46.21, 88.30)	60.08 (44.32, 79.91)	72.14 (52.65, 99.66)	<0.001*
TMT B^a^ (s)	82.00 (60.52, 118.71)	74.79 (54.15, 105.00)	102.43 (70.57, 139.76)	<0.001*
Stroop test A^a^ (s)	31.28 (25.50,38.76)	30.05 (25.00,35.21)	34.48 (27.24,41.15)	<0.001*
Stroop test B^a^ (s)	44.82 (33.78, 55.84)	41.55 (31.80, 52.58)	49.49 (37.99, 59.62)	<0.001*
Stroop test C^a^ (s)	92.90 (72.15, 115.37)	86.00 (70.00, 110.00)	104.49 (79.23, 125.86)	<0.001*
DSST^a^	27 (20, 33)	27 (23, 35)	24 (18, 28)	<0.001*
Maze^a^ (s)	39.30 (27.69, 51.82)	35.90 (26.27, 49.40)	42.27 (31.40, 58.51)	0.001*
DCT^a^	20 (15, 24)	21 (18, 26)	17 (12, 20)	<0.001*

CMB, cerebral microbleed; DCT, digit cancellation test; DSST, digit-symbol substitution test; MMSE, mini-mental state examination; TMT, trail making test; VF, verbal fluency.

^a^Median (quartiles).

*P < 0.05.

**FIGURE 1:**
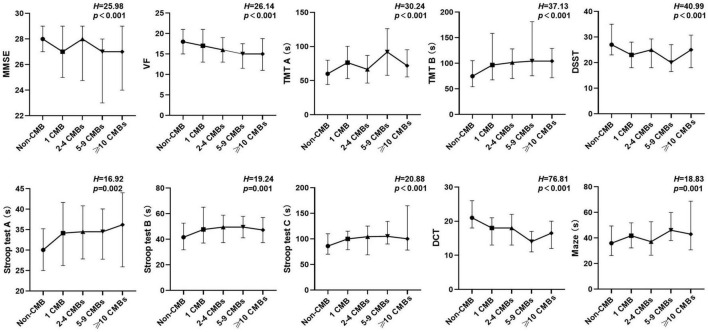
Comparison of cognitive function among subjects with different CMB burden grades (Kruskal–Wallis H test). CMB, cerebral microbleed; DCT, digit cancellation test; DSST, digit-symbol substitution test; MMSE, mini-mental state examination; TMT, trail making test; VF, verbal fluency.

All subjects were divided into five CMB burden grades according to the total number of CMB lesions. There were 345 non-CMB subjects, 67 patients with 1 CMB, 66 patients with 2–4 CMBs, 41 patients with 5–9 CMBs, and 44 patients with ≥10 CMBs. We also found the results of all cognitive function tests were significantly different among five CMB burden grades (MMSE, *p* < 0.001; VF, *p* < 0.001; TMT A and B, *p* < 0.001; Stroop test A, *p* = 0.002; Stroop test B, *p* = 0.001; Stroop test C, *p* < 0.001; Maze, *p* = 0.001; DSST, *p* < 0.001; DCT, *p* < 0.001). These comparisons are shown in Figure 1.

The results of Spearman correlation analysis showed the CMB number had positive correlations with the results of TMT (A, *rho* = 0.20; B, *rho* = 0.25; *p* < 0.001), Maze (*rho* = 0.16, *p* < 0.001) and Stroop test (A, *rho* = 0.17; B, *rho* = 0.18; C, *rho* = 0.18; *p* < 0.001). And, there were negative correlations between the CMB number and the performance of MMSE (*rho* = −0.21, *p* < 0.001), VF (*rho* = −0.21, *p* < 0.001), DSST (*rho* = −0.25, *p* < 0.001) and DCT (*rho* = −0.36, *p* < 0.001).

The multiple linear regression analysis showed after adjusting for age, gender and years of education (Model 1), there were significant positive correlations between the CMB burden grade and Stroop test (A, β = 0.10, *p* = 0.012; B, β = 0.09, *p* = 0.031; C, β = 0.19, *p* < 0.001), TMT (A, β = 0.13, *p* = 0.001; B, β = 0.09, *p* = 0.015) and Maze (β = 0.13, *p* = 0.001), and negative correlations between CMB burden and MMSE (β = −0.12, *p* = 0.001), VF (β = −0.16, *p* < 0.001), DSST (β = −0.12, *p* < 0.001) and DCT (β = −0.24, *p* < 0.001). Based on Model 1, smoking, hypertension, stroke/TIA, antihypertensive drugs, antithrombotic drugs, triglyceride, homocysteine, lacune, periventricular and deep WMH, and BG-PVS were added into the regression equation as Model 2. There were still significant correlations between the CMB burden grade and VF, Stroop test C, Maze and DCT (Table 3). However, the relationships between CMB burden and MMSE, Stroop test A, Stroop test B, TMT A, TMT B, and DSST were no longer significant (*p* > 0.05). There was no statistical difference in the incidence of posterior atrophy between CMB subjects and non-CMB subjects (Table 1). So, brain atrophy was not a covariate in the multifactor linear regression. However, when it was added to the regression equation, the results were consistent with Model 2. Brain atrophy was an excluded variable in the stepwise method.

**TABLE 3:** Linear regression analysis of the relationship between CMB burden grades and cognitive test results.

		Unstandardized coefficients *B*	Standardized coefficients β	95% CI	*p* for trend
VF	Model 1	−0.16	−0.16	−0.24, −0.09	<0.001*
	Model 2	−0.10	−0.10	−0.18, −0.02	0.019*
Stroop test C	Model 1	2.07	0.19	1.18, 2.96	<0.001*
	Model 2	1.42	0.13	0.48, 2.36	0.003*
Maze	Model 1	0.65	0.13	0.26, 1.03	0.001*
	Model 2	0.65	0.13	0.26, 1.03	0.001*
DCT	Model 1	−0.31	−0.24	−0.40, −0.22	<0.001*
	Model 2	−0.20	−0.15	−0.30, −0.10	<0.001*

CMB, cerebral microbleed; DCT, digit cancellation test; VF, verbal fluency 95% CI = 95% confidence interval. Model 1 was adjusted for age, sex and years of education. Model 2 was adjusted for age, sex, years of education, smoking, hypertension, stroke/TIA, antihypertensive drugs, antiplatelet drugs/anticoagulants, triglyceride, homocysteine, lacune, periventricular and deep WMH, and BG-PVS.

*P < 0.05.

As for the association between the results of cognitive tests and the number of CMB lesions, we found the CMB number is correlated positively with TMT (A, β = 0.10, *p* = 0.016; B, β = 0.13, *p* = 0.001) and Maze (β = 0.11, *p* = 0.006), and negatively with MMSE (β = −0.08, *p* = 0.040), VF (β = −0.10, *p* = 0.018), DSST (β = −0.12, *p* = 0.002) and DCT (β = −0.19, *p* < 0.001), after adjusting for all confounding factors (age, gender, years of education, smoking, hypertension, stroke/TIA, antihypertensive drugs, antithrombotic drugs, triglyceride, homocysteine, lacune, periventricular and deep WMH, and BG-PVS). There was no significant relationship between CMB number and Stroop test (*p* > 0.05).

## Discussion

In our study, CMB patients showed significantly worse performance in global cognitive function (MMSE) compared with the non-CMB group. CMB patients took much more time in Maze, TMT and Stroop test, and had fewer correct responses in VF, DSST, and DCT. Spearman correlation analysis showed the total number of CMB lesions had positive correlations with the TMT, Maze and Stroop test, and negative correlations with MMSE, VF, DSST, and DCT. After adjusting for age, sex and education, the CMB burden was significantly associated with the performances of all seven cognitive function tests. After further adjustment for all confounders, only the relationship of CMB burden with VF, Stroop test C, Maze, and DCT remained significant.

A growing number of studies have found that CMB is associated with vascular cognitive impairment. A meta-analysis of prospective studies showed that CMB patients were connected with overall 1.84 times increased risk of developing dementia than individuals without CMB, and CMB lesions increased the risk of progressing to incident dementia over time (Hussein et al., 2023). Over a mean follow-up of around 5 years, 1.5−4.5% of participants developed all-cause dementia, and 3.5−4.6% of CMB patients progressed to dementia (Akoudad et al., 2016; Ding et al., 2017).

Mini-Mental State Examination is the most well-known and widely utilized global cognitive function scale (Anderson, 2019). However, this scale is easily affected by age, education level and cultural background, and it is short of the evaluation item for executive function. Therefore, we used other tests focusing on different cognitive domains to conduct a more comprehensive assessment of cognitive performance. The Stroop test is commonly used as an indicator of attentional and executive measures. Stroop test A is related to the speed of visual search, Stroop test B is related to the working memory and visual search, and Stroop test C reflects working memory, conflict monitoring and visual search (Periáñez et al., 2021). The TMT is considered one sensitive standard of visual scanning, graphomotor speed and executive function. The score of TMT A can reflect the performance of visual scanning, graphomotor speed and visuomotor processing speed, while the TMT B and derived TMT scores are mainly related to working memory and inhibition control (Llinàs-Reglà et al., 2017). The VF is not only associated with language function but also describes the model of cognitive processes involved in task performance mainly: semantic memory access and executive function (Piskunowicz et al., 2013). Besides, good performances on the DSST, DCT, and Maze require the intact function of associative learning, motor control, complex attention, and visuoperceptual function (Jaeger, 2018; Canfora et al., 2021).

Some researchers combined the findings of several cognitive tests to represent the function of a specific cognitive domain, and supported the results that mixed CMB lesions and higher CMB burden mostly correlated with the dysfunction in global cognition, executive function, processing speed and memory (Akoudad et al., 2016; Ding et al., 2017; Gyanwali et al., 2021; Li et al., 2021). In this study, we described the performance of CMB patients on seven cognitive tests targeting different cognition domains. We found CMB burden grades were closely related to the results of VF, Stroop test C, Maze and DCT, but not MMSE, TMT (A and B), Stroop test A, Stroop test B and DSST, after adjusting for all covariates. These four tests had a better correlation with the severity of CMB, which may help clinical observation of the progression of CMB.

Some other studies also evaluated the performance of CSVD patients on different cognitive tests, but inconsistent results were found. A recent Chinese study used quantitative susceptibility mapping (QSM) to quantitatively measure the brain iron deposition burden of CMB lesions, which showed the iron deposition burden was one of the influencing factors for TMT (TMT A + TMT B) and MoCA scores (Li et al., 2022). A high-resolution MRI study found subjects with more than 10 CMBs showed significantly lower scores of VF than the non-CMB group, while there was no difference in TMT (Part A and B) between the two groups (Ueda et al., 2016). A community-based longitudinal study suggested strictly lobar CMBs (not deep or infratentorial) were related to MMSE and visuospatial executive function (Taylor complex figure test and clock drawing test) (Chung et al., 2016). However, another study demonstrated the number of lacunes was the main predictive factor of cognitive dysfunction in patients with cerebral autosomal-dominant arteriopathy with subcortical infarcts and leukoencephalopathy disease, while there was no significant association between CMB number and MMSE, TMT B, and Stroop test C (Lee et al., 2011). Differences among the findings may be caused by heterogeneities in the population, study design, operation procedure of cognitive tests and covariates in the regression equation.

The location of CMB lesions may have different effects on cognitive domains, and it has become one of the focus topics of CMB-related cognitive impairment. Some studies found only strictly lobar or lobar CMB was associated with cognitive impairment (Poels et al., 2012; Chung et al., 2016; Li et al., 2020), however, some different studies found a close relationship between deep, infratentorial or mixed CMB lesions and cognitive dysfunction (Qiu et al., 2010; Wang et al., 2019). Those inconsistencies may be due to the heterogeneities in study population, different types of cognitive tests, different grouping method of CMB locations. In our present study, the number of CMB patients in each location group is not large enough, especially in the strictly infratentorial sub-group. As a result, we did not analyze the relationship between the CMB location and cognitive function. More studies with large sample sizes will be needed urgently to quantify the CMB burden in different brain sub-regions.

The mechanism of cognitive dysfunction caused by CMB is still the theoretical speculation, which may be the direct damage of CMB to the surrounding brain tissue, disruption of white matter tracts and cortical-subcortical circuits, as well as effects on brain structure and functional networks (Ter Telgte et al., 2018; Jo et al., 2022; Nannoni et al., 2022). An animal experiment of inflammatory response after a cortical microhemorrhage has found CMB may impact the adjacent brain microenvironment through the migration and proliferation of brain-resident microglia and the activation of astrocytes (Ahn et al., 2018).

As for the management of CSVD-related cognitive impairment, it is centered on preventing and controlling vascular risk factors such as hypertension, diabetes, smoking and obesity. Symptomatic treatment includes cholinesterase inhibitors, N-methyl D-aspartate antagonist and ginkgo biloba. The other protective factors include higher education, occupation, social networks, good sleep quality, cognitive, and physical exercise (Zanon Zotin et al., 2021). Some studies have found that antiplatelet and anticoagulant drugs may increase the risk of CMB prevalence and intracerebral hemorrhage in CMB patients (Darweesh et al., 2013; Tsivgoulis et al., 2016). However, these drugs are crucial for the treatment of ischemic cerebrovascular disease. A recent study has found that cilostazol versus aspirin may be a better option in ischemic stroke with a high CMB burden (Park et al., 2021). More clinical trials are needed to provide more evidence for antithrombotic therapy in patients with CMB.

The highlight of this study was the use of multiple cognitive function tests to compare the cognitive characteristics between CMB patients and non-CMB subjects from multiple cognitive domains, and also describe the changes in cognitive performances with the severity of CMB in Chinese CMB patients. This study may provide a more theoretical basis for the selection of cognitive assessment scales for CMB patients in clinical practice.

There were some limitations in our study. Firstly, this was a single-center study based on one hospital, and there might be some selection, information or confounding bias. We recruited subjects strictly, enhanced standardized training of raters, and used multiple regression analysis to control potential confounders. However, the extrapolation of these conclusions still needed to be cautious. Secondly, according to previous studies, CMB lesions had more serious damage to the executive function, processing speed, attention and memory (Qiu et al., 2010; Poels et al., 2012; Akoudad et al., 2016; Ding et al., 2017; Li et al., 2021). So, we selected the commonly used scales for these cognitive domains. However, not all subjects completed the auditory vocabulary learning test (15 words), as a result, memory function was not included in our analysis. Thirdly, the number of CMB patients in different location groups is relatively small, and we did not analyze the relationship between the CMB distribution and cognitive scale results. Finally, the volume of CMB lesions has not been quantitatively evaluated, which can be improved using the QSM and other post-processing methods for the magnetic resonance image in the future.

## Conclusion

In our present study, subjects with CMB showed obvious abnormalities in global cognitive function, executive function, processing speed, attention and language. The cognitive battery we used, especially VF, Maze, DCT, and Stroop test, may be helpful to reflect the severity of CMB and play an auxiliary role in clinical practice. In future studies, quantitative analysis of CMB lesions and assessments for other cognitive domains should be carried out. Moreover, it’s essential to conduct more research on the underlying pathophysiology of CMB and the mechanism for CMB-related cognitive dysfunction.

## Data availability statement

The original contributions presented in this study are included in the article/supplementary material, further inquiries can be directed to the corresponding authors.

## Ethics statement

The studies involving human participants were reviewed and approved by the Ethics Committee of Beijing Chaoyang Hospital, Capital Medical University. The patients/participants provided their written informed consent to participate in this study.

## Author contributions

WH and JY contributed to the conception and design of the study. XL, SY, YL, and JY contributed to the data collection and database organization. XL performed the statistical analysis and wrote the first draft of the manuscript. JY wrote sections of the manuscript. LY and WQ helped with important intellectual content. All authors contributed to the manuscript revision, read, and approved the submitted version.
